# Objective Detection of Tinnitus Based on Electrophysiology

**DOI:** 10.3390/brainsci12081086

**Published:** 2022-08-16

**Authors:** Shuwen Fan, Shufeng Li

**Affiliations:** 1 Department of Otolaryngology and ENT Institute, Eye & ENT Hospital, Fudan University, Shanghai 200031, China; 2 NHC Key Laboratory of Hearing Medicine, Fudan University, Shanghai 200031, China

**Keywords:** tinnitus, electrophysiology, objective, waveform

## Abstract

Tinnitus, a common disease in the clinic, is associated with persistent pain and high costs to society. Several aspects of tinnitus, such as the pathophysiology mechanism, effective treatment, objective detection, etc., have not been elucidated. Any change in the auditory pathway can lead to tinnitus. At present, there is no clear and unified mechanism to explain tinnitus, and the hypotheses regarding its mechanism include auditory plasticity theory, cortical reorganization theory, dorsal cochlear nucleus hypothesis, etc. Current theories on the mechanism of tinnitus mainly focus on the abnormal activity of the central nervous system. Unfortunately, there is currently a lack of objective diagnostic methods for tinnitus. Developing a method that can detect tinnitus objectively is crucial, only in this way can we identify whether the patient really suffers from tinnitus in the case of cognitive impairment or medical disputes and the therapeutic effect of tinnitus. Electrophysiological investigations have prompted the development of an objective detection of tinnitus by potentials recorded in the auditory pathway. However, there is no objective indicator with sufficient sensitivity and specificity to diagnose tinnitus at present. Based on recent findings of studies with various methods, possible electrophysiological approaches to detect the presence of tinnitus have been summarized. We analyze the change of neural activity throughout the auditory pathway in tinnitus subjects and in patients with tinnitus of varying severity to find available parameters in these methods, which is helpful to further explore the feasibility of using electrophysiological methods for the objective detection of tinnitus.

## 1. Introduction

Tinnitus is an auditory phantom perception in the absence of acoustic stimulation. Its risk factors include hearing loss, ototoxic drugs, head injury, and depression. Persistent tinnitus will influence patients’ ability to socialize and communicate and will result in insomnia, anxiety, depression, and other psychological problems, and eventually reduce their quality of life [1]. The majority of studies show that the prevalence rate of tinnitus in adults is between 10% and 15% [2]. However, few errors perhaps exist due to the unclear definition of tinnitus and inappropriate epidemiological methods [3]. Tinnitus can be classified into two categories, subjective and objective tinnitus. Objective tinnitus, which is uncommon, can be heard not only by the patients themselves, but also can be perceived by the clinician. The majority of tinnitus patients suffer from subjective tinnitus, which can only be perceived by themselves. Objective detection methods of tinnitus are lacking due to the subjective nature of tinnitus, and diagnosis is mainly based on subjective measurements, such as self-report questionnaires, case history, pure tone audiometry, psychoacoustic testing, etc. Although there are irreplaceable advantages in diagnosing tinnitus through subjective reports at present, when it comes to psychological problems, insurance or pension benefits, the development of credible measurement methods for objective diagnosis of tinnitus can provide professional judgment. In addition, when classifying tinnitus and judging the efficacy of drugs, an objective index is more helpful to the judgment of experimental results [4]. Moreover, prelingual children and patients with low-functioning behavioral abilities or cognition [5] need to be tested by the objective method.

The underlying mechanism of tinnitus is not yet clear, it is likely to result from a maladaptive neuroplastic response to sensory deprivation [6]. The “central gain” theory [7] holds the point that when the electrical activity afferent to the cochlea decreases or disappears, the central auditory pathway compensates for this deficiency through the increase in spontaneous firing rate and synchronous activity of neurons, such as the cochlear nucleus and inferior colliculus, which increases the synaptic efficiency of auditory central neurons and produces tinnitus [8]. The central auditory regions mistakenly interpret abnormal nerve activity as the sound result of tinnitus, thus the use of electrophysiological tests to evaluate the related nerve activity of tinnitus has become one of the most widely used methods in the clinic. Meanwhile, modulation of non-auditory processes from limbic and paralimbic structures is also involved. According to the gating mechanism, individual differences in the effectiveness of structure-mediated noise cancellation systems within the non-auditory area play a decisive role in the presence or absence of tinnitus [9]. In addition, Llinás et al. proposed a model of thalamo-cortical dysrhythmia, in which deafferents cause interruption of activity between the thalamus and cortex, leading to hearing loss, which leads to inhibition of thalamic neurons. This, in turn, results in oscillatory activity at the level of the cerebral cortex and changes in large-scale slow-wave and gamma activity in adjacent cortical regions, leading to tinnitus [10]. The Bayesian brain model proposes that the brains of subjects with peripheral hearing loss continuously generate predictions regarding the environment to reduce sensory uncertainty caused by a limited amount of auditory information, based on auditory predictions and the resulting prediction error, which can cause tinnitus [11]. 

Unfortunately, there is still no unified pathophysiological mechanism of tinnitus. The main reasons include the inability to objectively detect the existence of tinnitus, the diversity of causes of tinnitus, and the influence of accompanied diseases of tinnitus. Current methods for objectively detecting tinnitus also include imaging, such as CT and MRI. However, a large amount of subjective tinnitus have no clear changes in imaging due to the problem of the resolution and the complex anatomical structure of the head. In addition, numerous theories of tinnitus mechanism involve electrophysiological changes, thus it has great potential to use electrophysiological methods to detect tinnitus objectively. Electrophysiological technology refers to the method of measuring, recording, and analyzing the electrical phenomena and characteristics of organisms by stimulating organisms with energy in the form of electricity, sound, etc. This type of measurement has little damage to the human body and is of great significance for clinical diagnosis. However, there is great heterogeneity in different studies when using some electrophysiological indicators to compare the tinnitus group with the control group. Although few methods were widely accepted in animal studies, there are several limitations when used in humans due to differences in their etiology and characteristics of tinnitus, the anatomy, and physiology of the auditory pathway. In addition, back to the original question, we are not sure whether the animal does have tinnitus since there are still some defects and deficiencies in the existing detection methods in animals. Finding a reliable objective evaluation method of tinnitus is crucial for the progress of tinnitus research. Therefore, in this review, we categorized objective methods related to the evaluation of tinnitus based on the results of published studies on electrophysiology examination, exploring the changes of components of the potential waveform recorded when tinnitus was measured by various methods, such as auditory brainstem response (ABR), event-related potential (ERP), gap detection, and electrocochleography (EcochG) to identify the work performed at present, discuss the limitations of these methods, ongoing challenges in the fields, and explore the direction of future research.

## 2. Electrophysiological Methods

### 2.1. ABR

#### 2.1.1. Components

The ABR, a technique recorded from the scalp that reflects the activity of nerve fibers in the auditory nerve and brainstem, is a powerful and most common objective diagnostic tool routinely used in clinical practice to determine hearing thresholds of infants, young children [12], and adult patients who are difficult to undergo behavioral testing [13]. In addition, it is a cost-effective clinical tool for identifying retrocochlear lesions, acoustic neuromas, and vestibular schwannomas [14,15]. ABR typically consists of five waves that occur during the first 10 ms after the presentation of a transient sound, and previous studies indicated that waves I, II, III, IV, and V generally represent the responses of neuronal activity in the spiral ganglion cells of the auditory nerve, cochlear nucleus, superior olivary complex, the medial superior olive and its projections to the nuclei in the lateral lemniscus, and inferior colliculus, respectively in cats [16], rats [17,18], and pinnipeds [19]. Recently, ABR has been proven to recognize cochlear synaptopathy, which is considered to be a mechanism of tinnitus [20]. Therefore, we can assess tinnitus according to electrical activity in the auditory pathway by observing the changes in the waveforms, and abnormal alterations in ABR can indicate the pathologic site of tinnitus. To date, a large amount of research works have been carried out and several papers have been published to explore the relationship between tinnitus and the morphology of ABR waveforms. 

#### 2.1.2. ABR Studies

When studying the relationship between tinnitus and ABR waveform, some studies have found changes in ABR wave amplitude and latency, such as prolonged latency of waves I [21] or V [22] or both [23,24]. Few studies reported no statistically significant difference in amplitude or latency between the tinnitus group and the control group [25,26,27,28]. Characteristics of these studies are shown in Table 1. The reduced amplitude of wave I, which was induced by deficiency of sensory input and enhanced ABR wave V/I amplitude ratio in tinnitus, was found in the majority of studies [29,30,31,32]. However, few studies have found that the wave V amplitude and V/I amplitude ratio decreased in the tinnitus group [33,34,35]. As mentioned above, the ABR composition of tinnitus patients varies greatly in different studies, and one of the main reasons for these different conclusions is the heterogeneity of the subjects. Studies have shown that gender, age, hearing status, and tinnitus duration all have an impact on the waveform of ABR [36,37,38,39]. Although the age, gender, and hearing status of the tinnitus group and the control group were matched in the majority of studies, the participants in the majority of studies included two types of genders. Moreover, there are differences in the age of the subjects and the definition of normal hearing among the studies, which may also lead to biases in the results. The majority of studies did not measure the hearing threshold in the frequency range of 8–16 kHz when including subjects with normal hearing. However, few studies have shown that most tinnitus patients with normal hearing have a high threshold at high frequency [40,41] and have confirmed that hearing threshold of high frequencies will affect the waveform of ABR [42]. Moreover, it was reported that audiometric configuration affected the ABR waveform in a study [43]. The relative contribution of the low frequency region of the cochlea to the wave V in ABR response is greater than the I wave. The high frequency damage may lead to the increase in the V/I amplitude ratio by increasing the influence of the low frequency region [44]. A study showed that within-subject ABR amplitudes are more reliable than between-subjects ABR amplitudes, indicating that different grouping methods will also have an impact on ABR waveform [45]. In two within-subject comparative studies, no change in ABR amplitude was found between the tinnitus side and the contralateral side [46,47], but one [48] of the studies found that the interpeak latency of III–V was prolonged. However, the etiology of tinnitus in the majority of studies is not reported. When grouped according to the etiology of tinnitus, homogeneous results are more likely to be obtained. There was no significant difference in the latency or amplitude of ABR waveforms between noise-induced tinnitus and control groups in a few studies [25,26]. This result may be attributed to the uncertainty of the amount of noise exposure in humans during their lifespan. However, there are problems, such as not ruling out hyperacusis, and the location of tinnitus does not match. The majority of experiments performed measurements at PTA below 8 kHz, and one study measured up to 16 kHz found a reduction in wave I in the tinnitus group. However, this study did not address the etiology of tinnitus and rule out hyperacusis [29]. Another study that also measured high-frequency hearing thresholds did not find statistically significant changes in ABR. Moreover, it did not rule out hyperacusis [26]. Of the few studies that excluded hyperacusis, one study found increased wave V and interpeak III–V latency only at 80 dB [22], and two other studies found reduced wave V amplitude [33,34]. However, there are one or more defects, such as unmeasured high-frequency hearing threshold and unknown etiology. In addition, the frequency, nature, and hearing level of stimuli during ABR measurements varied across studies. Overall, the reduced amplitude of wave I, which was induced by deficiency of sensory input and enhanced ABR wave V/I amplitude ratio in tinnitus, was found in the majority of studies [29,30,31], and variations in the parameters of the waveform in ABR exist between different studies. As previously mentioned, changes in the auditory nerve are reflected in wave I. Therefore, the reduced amplitude of wave I indicates the reduced sensory input found in tinnitus and represents the damage of the synaptic ribbons of the inner hair cells and spiral ganglion cells [20,49,50]. Similarly, the elevation in ABR wave V amplitude indicates central hyperactivity in the lateral lemniscus and inferior colliculus [30,49]. However, other studies [29] found that the amplitude of wave V is normal, which probably resulted from the compensation for the reduced activity in the auditory nerve by increasing the neural responsiveness of the central auditory system, and the elevated wave V is caused by the use of a lower frequency filter cut-off. As mentioned above, changes in amplitude correspond with the central gain theory, in which the enhanced ABR wave V/I amplitude ratio is considered a potential objective indicator for diagnosing tinnitus [29,30,31,44,51]. However, few recent studies have shown that the gain increases may be irrelevant or even protective to tinnitus, while about 50% of tinnitus also suffer from hyperacusis [52], which can lead to changes in ABR waveforms and confuse the results [33,53,54]. In addition, one study [33] found that the increased IV/I (animal) or V/I (human) amplitude ratio and prolonged latencies of ABR waves are part of a healthy, dynamically balanced adaptation process in subjects without symptoms. The reduction rather than enhancement of IV or V amplitude of ABR wave may be a potential biomarker of tinnitus in animals or patients without co-occurrence of hyperacusis [34,53]. They believe that the co-occurrence of hyperacusis is one of the important reasons why the previous studies are different from theirs. In addition, when tinnitus is combined with hyperacusis, the patient will not be able to tolerate the high level of ABR measurement [55]. Moreover, reduced amplitude and prolonged latency of ABR may be related to deafferentation of high spontaneous auditory nerve fibers, which show low response thresholds. Therefore, an appropriate compensatory increase in the IV or V of the ABR wave is hindered since a sufficient increase in discharge rate is not generated to compensate for the deprived input. Recently, a study shows that due to low signal-to-noise ratio and individual variability, the application of amplitude or slope of wave I in the diagnosis of human tinnitus is limited [55]. In addition, the duration of tinnitus has some influence on ABR waveform, and we should try our best to eliminate this heterogeneity in future research [36]. Moreover, compared with the control group, the waveform of click evoked ABR in tinnitus patients was different from the tone burst evoked ABR [56], and in patients with bilateral tinnitus, the wave patterns of left and right ears are different [57]. Furthermore, abnormal ABR is more prominent in tinnitus of multiple features. Specifically, when there is more than one type of pitch in patients with tinnitus, there is also a significant difference in ABR compared with patients with single feature tinnitus [58]. 

Several factors that affect ABR need to be explored including detailed normal variations in tinnitus of different genders, ages, etiology, hearing status, localization, tinnitus duration, and stimulus patterns. Moreover, as many variables as possible need to be controlled in future studies.

### 2.2. ERP

#### 2.2.1. Components

Event-related potentials, averaging signals extracted from the electroencephalogram (EEG) recorded by electrodes on the scalp, are related to physical or psychological events in time. In addition, they are generally used to detect the discharge time of auditory pathway fibers, and to indicate the presence of neuronal activity [59]. Moreover, they can show the functionality and connection between different areas of the brain using multi-channel recording systems [60], and distinguish cognitive processes in pathological states from the normal human brain [61]. ERP can be divided into P1, N1, P2, N2, and P3 according to their peak latencies. In addition, the mismatch negativity (MMN) which is a pre-attentive auditory ERP will be discussed in this review. P1, which generally evoked 50–100 ms after the stimulus, is considered to be the sensory gating, filtering the stimulus and switching passive attention [62]. Frequent stimulus without change will evoke N1, P2, and N2 peaks, which are the sensory potential, representing the involuntary attention [63,64], and the range of latencies of these peaks are 100–200 ms, 180–280 ms, and 225–350 ms, respectively [62]. When a rare stimulus is raised, P300 will be generated [65]. The first three waves, showing negative-positive-negative polarities, respectively, were defined as the N1–P2–N2 complex [66]. Moreover, the P3 auditory response is typically generated from the areas of the hippocampus, superior temporal sulcus, the ventrolateral prefrontal cortex, and the intraparietal sulcus of the brain probably [67,68,69,70], and is named after a large and positive peak, which is induced 300 ms after the stimulus and approximately range from 300 to 400 ms [71]. The N1–P2 can detect almost any discriminable change in the auditory environment [72], and represents the late phase of sensory gating [73] as well as sensitive indexes of the degree of arousal or wakefulness [74]. Meanwhile, the N1 can reflect the recognition of stimuli [63] and short-term memory [75]. Moreover, it relates to sound detection and attention-catching properties [76,77]. The main neural generators of N1 are primary [78] and secondary [79] auditory cortices. While one study assumed that P2 can identify a stimulus as a target, only in this way can P300 be elicited subsequently. Therefore, P2 can classify stimuli by some means. In addition, the prominent brain region of this function is involved in preventing interference from irrelevant stimuli [74]. Furthermore, primary processes of allocation of attention can be recorded by P2 [80]. The N2 in the frontal cortex is linked to human inhibitory control [81] and is thought to reflect activation of the anterior cingulate cortex [82] and frontal lobe [83]. The amplitude of P3 will change with the change of attention [84] and perception [85]. The majority of studies focus on the association between auditory P3 and selective auditory attention ability, while only a few studies examined auditory P3 in tinnitus patients. MMN, elicited by any discriminable change of auditory stimulation and occurring with a latency ranging from 100 to 250 ms, is considered to distinguish the incoming stimulus from the sensory trace of the preceding stimulus [61]. More precisely, MMN is a separate negative deflection which responds to the deviant stimulus, and the MMN will be larger and earlier when the gap between deviant and standard stimulus becomes wider, leading to MMN and N1 overlap [63]. To measure the MMN amplitude precisely, quantifying should be performed after the N1 latency window to avoid contamination by possible differences in the N1. Moreover, MMN is considered to be the best objective measure to evaluate discrimination acuity since even threshold-level differences can elicit it. The applications of clinical disorders of MMN contain coma, schizophrenia, cognitive decline, and dyslexia in children [61]. Converging evidence supports the notion that MMN reflects the attention switching triggered by the automatic auditory change detection process [62].

**Table 1:** Characteristics of ABR studies, including demography, stimulation patterns, and outcomes.

Author, Year	Groups and Number	Etiology of Tinnitus	Mean Age	Matched	Hearing Status	Hyperacusis	Stimuli	Outcome (Tinnitus Group)
Schaette and McAlpine, 2011 [29]	15 tinnitus 18 controls	not mentioned	tinnitus: 36.3 controls: 33.2	age, sex, hearing	≤20 dB HL (0.125–8 kHz, 12–16 kHz)	not mentioned	duration: 50 µs, 90 and 100 dB SPL, 11 clicks/s	reduced wave I amplitude and normal wave V
Singh et al., 2011 [21]	25 tinnitus 20 controls	idiopathic	tinnitus: 32 controls: matched	age, sex, hearing	<25 dB HL (0.25–8 kHz)	not mentioned	not mentioned	prolonged wave I latency, shortened wave V, and I–III and I–V interpeak latencies
Cartocci et al., 2012 [22]	10 tinnitus 14 controls	idiopathic	tinnitus: 43.9 controls: 45.1	age, sex, hearing	≤20 dB HL (0.125–8 kHz)	excluded hyperacusis by the dynamic range measure	alternating polarity, duration: 100 μs, 90 and 80 dB HL, 11 clicks/s	prolonged wave V and interpeak III–V latencies
Gu et al., 2012 [30]	15 tinnitus 21 controls	not mentioned	tinnitus: 42 controls: 43	age, sex, hearing	≤20 dB HL (0.125–8 kHz)	not mentioned	condensation, duration: 100 μs, 30, 50, 70, and 80 dB HL, 11 clicks/s	reduced wave I amplitude and enhanced wave V amplitude
Nemati et al., 2014 [31]	25 tinnitus 16 controls	idiopathic	tinnitus: 34.4 controls: matched	age, sex, hearing	<25 dB HL (0.25–8 kHz)	not mentioned	alternating polarity, duration: 90 dB SPL, 11.1 clicks/s	enhanced V/I amplitude ratio
Santos-Filha et al., 2014 [25]	30 tinnitus 30 controls	noise induced	tinnitus: 41 controls: 41.6	age, sex, hearing	<25 dB HL (0.25–8 kHz)	not mentioned	Rarefaction polarity, duration: 0.1 ms, 80 dB HL, 19 clicks/s	no significant differences in latencies
Gilles et al., 2016 [26]	19 tinnitus 23 controls	noise induced	male 23.1 female 23.5	age, sex, hearing	<25 dB HL (0.125–8 kHz, 9–16 kHz)	3 subjects of the non-tinnitus group and 4 subjects of the tinnitus group had a score >22 on the hyperacusis questionnaire	alternating polarity, duration: 100 µs, 80 dB HL, 31 clicks/s	no significant differences
Konadath et al., 2016 [35]	20 tinnitus 20 controls	idiopathic	tinnitus: 33.15 controls: 20.50	sex, hearing	≤20 dB HL (0.25–8 kHz)	not mentioned	duration: 100 µs, 70 dB HL, 11.1 clicks/s	reduced absolute amplitude of peaks I and V
Kehrle et al., 2016 [86]	84 tinnitus 47 controls	not mentioned	tinnitus: 37.2 controls: 35.7	age, sex, hearing	≤25 dB HL (0.25–8 kHz)	not mentioned	negative polarity, duration: 100 ms, 80 dB HL, 21.1 clicks/s	abnormal values for the latency of wave I, wave III, wave V, the interpeak I–III, the interpeak III–V, and the interpeak I–V
Ravikumar et al., 2016 [24]	50 tinnitus 50 controls	not mentioned	not mentioned	not mentioned	not mentioned	not mentioned	not mentioned	prolonged I, III, and V
Shim et al., 2017 [46]	43 tinnitus 18 controls	not mentioned	tinnitus: 33.6 controls: 28.6	age, sex, hearing	≤20 dB HL (0.25–8 kHz)	not mentioned	duration: 90 dB HL, 13.3 clicks/s	no significant differences
Guest et al., 2017 [27]	20 tinnitus 20 controls	noise induced	tinnitus: 25.7 controls: 25.5	age, sex, hearing	≤20 dB HL (0.25–8 kHz)	not mentioned	duration: 102 dB ppe SPL (peak-to-peak), 14.1 clicks/s	no significant differences in amplitude of wave I and V
Pinkl et al., 2017 [56]	11 tinnitus with 21 tested ears 10 controls with 10 ears	idiopathic	tinnitus: 46.48 controls: 24.4	not mentioned	<30 dB HL (0.25–20 kHz)	not mentioned	click evoked ABRs: rarefaction polarity, 85 dB HL, 21.1 stimuli/s tone burst evoked ABRs: rarefaction polarity, 85 dB HL, 21.1 stimuli/s	Click ABR: prolonged V–III IPLs for tinnitus with normal hearing and tinnitus with hearing loss and prolonged absolute V latency for tinnitus with hearing loss. Tone burst ABRs: prolonged absolute latencies and IPLs at three of the seven frequencies for tinnitus with hearing loss
Bramhall et al., 2018 [44]	15 tinnitus 59 controls	noise induced	tinnitus: 26.3 controls: 26.7	age, hearing	≤20 dB HL (0.25–8 kHz)	pure tone loudness discomfort levelswere measured as an indicator of hyperacusis	alternating polarity, duration: 4 kHz tone burst stimuli, duration: 2 ms, 80, 90, 100, and 110 dB ppe SPL, 11.1 stimuli/s	reduced wave I amplitudes and wave I/V ratio
Song et al., 2018 [57]	20 tinnitus 91 controls	not mentioned	tinnitus: 37 controls: 43	age, sex, hearing	≤20 dB (0.25–8 kHz)	not mentioned	duration: 90 dB click stimulus	shortened latency in wave III on the right and in wave V on the left in patients with bilateral tinnitus
Hofmeier et al., 2018 [53]	17 tinnitus 17 controls	idiopathic	tinnitus: 33.2 controls: 36.5	sex, hearing	≤40 dB (0.125–10 kHz)	excluded by the Hyperacusis Questionnaire	duration: 100 µs, 25–75 dB SPL in 10 dB steps, 11.1 clicks/s	reduced and prolonged wave V
Majhi et al., 2019 [23]	55 sensorineural hearing loss with tinnitus 51 control	idiopathic	tinnitus: 42.91 controls: 41.63	age, sex, education level	Sensorineural hearing loss mild = 26–40 dB, moderate = 41–60 dB, severe >61 dB	not mentioned	not mentioned	prolonged latency of wave I, III, V, and interpeak latency of I–III, III–V, I–V was observed in tinnitus with sensorineural hearing loss group
Möhrle et al., 2019 [33]	17 Tinnitus 17 Controls	not mentioned	not mentioned	All standard conditions	PTA ≤40 dB	excluded by the new Hyperacusis Inventory Questionnaire	duration: 0.1 ms, 25–75 dB SPL in 10 dB steps,11.1 clicks/s	prolonged latencies and reduced amplitudes of ABR wave V
Han et al., 2021 [48]	10 tinnitus 6 chronic tinnitus 4 non-chronic tinnitus	idiopathic	tinnitus: 16.5		≤25 dB HL (0.125–8 kHz)	not mentioned	duration: 90 dB, 13.3 clicks/s	prolonged interpeak latency of III–V in tinnitus ears compared with non-tinnitus ears
Hofmeier et al., 2021 [34]	43 controls 30 tinnitus 20 tinnitus + hyperacusis	not mentioned	not mentioned	not mentioned	not mentioned	hyperacusis questionnaire	not mentioned	reduced ABR wave V amplitude, prolonged interpeak latency (IPL) I–V, reduced ABR wave V/I ratios
Shim et al., 2021 [47]	27 tinnitus 27 controls	idiopathic	tinnitus: 36.7 controls: 36.0	age, sex, hearing	≤20 dB HL (0.25–8 kHz)	not mentioned	duration: 100 ms, 80 and 90 dB HL, 13 clicks/s	no significant differences in wave I or wave V or the wave V/I
Johannesen et al., 2021 [28]	7 tinnitus 87 controls	not mentioned	not mentioned	not mentioned	≤20 dB HL at 0.5 and 4 kHz and ≤30 dB HL at 6 and 8 kHz	not mentioned	rarefaction clicks, duration: 100 μs, intensities: from 110 down to 90 dB, peak-to-peak equivalent sound pressure level (ppe SPL), in 5-dB steps rate: 11 clicks/s	no significant differences in wave I and V amplitudes
Park et al., 2021 [32]	59 tinnitus 59 controls	idiopathic	tinnitus: 42.42 controls: 41.86	age, hearing	≤25 dB	not mentioned	clicks, duration: 50 ms, 90 dB SPL	reduced ABR wave I amplitude and wave I/V ratio
Sendesen et al., 2022 [87]	20 unilateral tinnitus	idiopathic	unilateral tinnitus: 33.55	sex, hearing (up to 16 kHz)	<20 dB HL (0.5–4 kHz)	not mentioned	alternating polarity, duration: 80 dB HL level, 21.1 clicks/s	enhanced wave I amplitude and the ratio of III/I, V/I, and V/III wave amplitude in tinnitus ears

#### 2.2.2. ERP Studies

To objectively assess tinnitus, several studies on the difference in the amplitude and latency of the waves in ERPs between tinnitus patients and controls have been reported in these years. The characteristics of these studies are summarized in Table 2. However, there is still no consensus regarding the alteration of these waves in tinnitus. One study showed that there was no difference between tinnitus and controls in P1 potential [88]. In addition, another study [60] found that after an effective treatment, P1 did not change, indicating that there is no defect in the function of sensory gating in tinnitus. The majority of studies showed that there was an alteration in the amplitude and/or latency of N1. The N1 amplitude will be lower in tinnitus patients compared with control subjects in the majority of studies [89,90,91], and the tinnitus group presented a larger N1 after an effective treatment [65], while the latency of N1 is increased [92,93]. These alterations of N1 may indicate the difficulty in switching attention from the tinnitus sound to external stimuli in patients [73]. However, few studies take a different view. In addition, increased N1 response has been found in a few studies [94,95,96], which corresponds to abnormal center gain. Moreover, increased neural synchronization or neuronal maladaptation are possible causes. Shortened N1 and P2 latencies in tinnitus patients in one study can be explained by the hyperexcitability of thalamocortical circuits [97]. Concerning P2, reduced amplitudes [89] and higher latencies [93,98] have been found in tinnitus patients in several studies. Increased latency of P3 without amplitude change was found in tinnitus patients in the majority of studies [73,93,99], while other studies found that the amplitude of P3 in tinnitus patients was significantly lower than in controls and there is no difference in latency [89,91,92,100,101]. Furthermore, larger P3 amplitudes along with prolonged latency were observed in individuals with tinnitus [96]. The impaired adaptive capacity of cortical neurons, wider firing range of neurons, and changes in the baseline activation of neurons leading to hyperexcitability of neurons can all lead to P3 enlargement [96]. Attention deficit due to the presence of tinnitus that prevents the patient from focusing on the stimuli provided may be responsible for the prolonged P3 latency [99,102]. Other studies have found that the P3 amplitude is reduced with a prolonged latency [103,104] or only the amplitude is increased [105]. A decrease in the number of working neurons, a decrease in neural activity or a mismatch in neuronal firing may be responsible for the reduction in P3 amplitude [93,103]. In addition, the tinnitus group before the treatment has lower MMN amplitude, while the group after the treatment shows larger MMN [65]. Moreover, another study found that the group who experienced tinnitus annoyance has lower MMN latency than the control group who do not have tinnitus [106]. Furthermore, numerous studies, including those abovementioned, found that some components of the ERP did not differ in amplitude or latency in tinnitus patients compared with controls. This may be related to subclinical deficits in central auditory processing skills, such as related deficits in hearing condition, attention, and arousal [96,107]. At present, P3 among the components of ERP has great potential as an indicator for objectively predicting tinnitus. Reduced P300 amplitude and prolonged latency may be due to dysregulation of synchronized activity of multiple discrete underlying neural structures [73].

Furthermore, a study examined whether the acoustic change complex (ACC) during changes in external acoustic stimuli could be used as an objective indicator of tinnitus. The basic theory is that if acoustic changes occur at the frequency of tinnitus, tinnitus may interfere with the detection of these changes. In this study, the demographic data, hearing threshold, and intensity stimulation levels of the tinnitus group and the control group were matched. In addition, ABR detection was carried out before the experiment to exclude patients whose waveforms changed. In the experiment, the tinnitus frequency in the tinnitus group was 8 kHz. The ratio of the N1′–P2′ complex wave amplitude evoked by the changing stimulus of 8 kHz or 4 kHz and the N1–P2 complex wave amplitude evoked by 1 kHz background stimulation was compared between the tinnitus group and the control group. The ratio was recorded as the normalized amplitude of the ACC. The results showed that the normalized amplitude of the ACC of 8 KHz (tinnitus frequency) in the tinnitus group was less than 4 and 8 kHz in the normal control group, and the normalized amplitude of the ACC of 4 kHz was higher than 8 kHz in the tinnitus group. However, whether other frequencies of tinnitus are applicable to patients remains to be explored [108]. Subsequently, Sedley et al. [109] found that intensity mismatch asymmetry (IMA) may be a potential biomarker of tinnitus. When the stimuli intensity was altered between 0 and 6 dB, both pure tone stimuli of frequencies at the tinnitus match center and at the lower edge of the Hanning passband showed that MMN responses to intensity deviants are asymmetry, namely, tinnitus subjects had larger responses to upward deviants and fewer responses to downward deviants than matched controls. The area under the ROC curve for the metric calculated from upward deviants minus downward deviants is 0.77. More importantly, this metric does not rely on highly specific tinnitus matching. It seems that focusing on changes in indicators is more likely to yield meaningful results.

#### 2.2.3. Main Findings

Decreased amplitudes and increased peak latencies have been observed in various psychiatric syndromes [110,111]. Neurological and psychological disorders are best left out when conducting experiments on ERP. In addition, medications that act on the central nervous system, such as serotonin reuptake inhibitors, can affect ERP [112]. Studies have shown that age and hearing conditions can affect ERP, and future experiments should pay attention to matching these two factors in the tinnitus group and the control group [103,113]. Moreover, the severity of tinnitus is a contributing factor, and Wang et al. [114] found that patients with severe tinnitus had longer N2 and P3 latencies than those with mild tinnitus. The majority of the experiments did not control for the duration of tinnitus in the tinnitus group, resulting in large differences within the group. However, few studies have shown that the duration of tinnitus affects the gamma network or executive attention, which may probably have an impact on ERP [115,116]. Numerous studies have confirmed that ERP is affected by “stimulus” and “electrode” factors [92]. The majority of studies used the auditory oddball paradigm, while some used pure tone stimuli [94]. In addition, some used gap-inserted stimuli, which will be discussed in “gap detection”. The oddball paradigm manifested as two different random stimuli with different frequencies. Despite the oddball paradigms used, the duration, frequency, the interval between the two stimuli, number of recording electrodes, location of recording electrodes, number of channels, etc., varied across experiments. A unified standard is yet to be developed.

In general, although there is still no consensus on the specific alteration of ERP in the tinnitus group, ERP components do indeed change. There has been a change in the way the auditory system perceives and processes sound in patients with tinnitus probably. The heterogeneity of previous studies may result from the difference in different studies, such as the subtype, severity, location of tinnitus, hearing condition, frequency of hearing loss, psychologic status, stimulation paradigm, etc. In addition, ERP is mainly used to evaluate advanced mental activities of the brain, such as cognitive process and attention. Therefore, when ERP is used to evaluate tinnitus, it is greatly influenced by these factors. It may be one direction to compare the baseline potential levels between the tinnitus group and the control group using some algorithm to filter the influence of these factors. Future studies on ERP should take these factors into account to explore the fundamental neurobiological mechanisms of tinnitus and how to make a definite diagnosis of tinnitus using ERP.

**Table 2:** Characteristics of ERP studies, including demography, stimulation patterns, and outcomes.

Author, Year	Groups and Number	Etiology of Tinnitus	Mean Age	Matched	Hearing Status	Neurological and/or Psychological Disorder	Electrode	Stimuli	Outcome (Tinnitus Group)
Santos Filha and Matas, 2010 [93]	30 tinnitus 30 controls	noise-induced	tinnitus: 41 controls: 41.6	age	≤25 dBHL (0.25–8 kHz)	excluded	right and left ears (A2 and A1); vertex (Cz) and forehead (Fpz)	oddball paradigm: tone bursts at 75 dB HL, in the frequencies of 1 kHz (frequent stimulus) and 1.5 kHz (rare stimulus)	prolonged latency of N1, P2, and P300
Gabr et al., 2011 [99]	40 tinnitus 40 controls	idiopathic	tinnitus: 37.3 controls: 38.5	age, sex, hearing	≤25 dBHL (0.25–8 kHz)	excluded	Fz (active electrode); Fpz (ground); M1 and M2 (reference)	oddball paradigm, in the frequencies of 1 kHz (standard stimulus) and 2 kHz (deviant stimulus)	prolonged P3 latency
Said, 2012 [104]	study group: 36 sensorineural hearingloss with tinnitus 30 sensorineural hearing loss only 24 controls	idiopathic	study group: 28.35 controls: 29.72	age, sex	heterogeneous	excluded	Fz (active electrode); Fpz (ground); M1 and M2 (reference)	80 dB HL, in the frequencies of 1 kHz (frequent stimulus) and 2 kHz (rare stimulus)	reduced P2 and P3 amplitude and prolonged N1, P2, P3 latency in patients with tinnitus
Elmorsy et al., 2013 [101]	32 tinnitus 30 controls	idiopathic	tinnitus: 39.8 controls: 38.7	age, sex, hearing	≤25 dB HL (0.25–8 kHz)	excluded	Fz (active electrode); A2 and A1 (reference); forehead (ground)	75 dB HL, in the frequencies of 1 kHz (frequent stimulus) and 2 kHz (rare stimulus)	overall reduced P3 amplitude and no significant differences for P3 latency
Holdefer et al., 2013 [106]	25 tinnitus 13 controls	not mentioned	tinnitus: 49 controls: 35	sex, hearing	tinnitus group’s mean PTA: 8 dB control group’s mean PTA: 9 dB	not mentioned	vertex (vertex electrode); behind the ears (right and left ear); the left side of the forehead (ground)	70 dB, in the frequencies of 1 kHz (standard stimulus) and 1.1 kHz (rare stimulus)	smaller latencies in the right ear, no statistically significant differences in MMN amplitudes
Yang et al., 2013 [65]	20 tinnitus 16 controls	not mentioned	tinnitus: 43.2 controls: 42.5	age, sex	tinnitus: PTA <20 dB (*n* = 8) PTA: 21–40 dB (*n* = 12) controls: PTA <20 dB (0.5, 1, 2, 4 KHz)	not mentioned	128 channels; Cz (reference channel); reported at Fz	oddball paradigm: pure tone at 75 dB, in the frequencies of 1500–1000 Hz (50-ms duration with a shaped 5-ms rise and fall time)	smaller mismatch negativity (MMN) and late discriminative negativity (LDN). After rTMS treatment, increased N1 response to deviant stimuli and larger MMN and LDN
Houdayer et al., 2015 [97]	17 tinnitus 17 controls	not mentioned	tinnitus: 43.4 controls: 45.7	not mentioned	<15 dB HL (0.125–8 kHz)	excluded	29 electrodes cap, obtained from the electrode displaying the greatest ERP	oddball paradigm: tone bursts, in the frequencies of 1 kHz (frequent stimulus) and 2 kHz (rare stimulus)	shorter N1 and P2 latencies. P300 did not differ between groups
Hong et al., 2016 [91]	15 tinnitus 15 controls	idiopathic	tinnitus: 30.2 controls: 28.7	age, sex	≤25 dB HL (0.25–8 kHz)	excluded	32 electrodes; the tip of the nose (reference); Afz (ground)	oddball and passive listening paradigm	shorter N2, lower N200 amplitudes during the oddball task compared with the passive listening task, lower P3. Lower N1 response to the target stimuli in the oddball task
Konadath et al., 2016 [35]	20 tinnitus 20 controls	idiopathic	tinnitus: 33.15 controls: 20.50	sex, hearing	≤20 dB HL (0.25–8 kHz)	not mentioned	2 channels; vertical (Fpz, Cz, M1/M2)	Alternating polarity 70 dB HL 500 Hz tone bursts 1.1/s	no significant difference in the latency and amplitude except for enhanced amplitude of the P1 peak in tinnitus group
Gopal et al., 2017 [94]	10 tinnitus 10 controls	heterogeneous	tinnitus: 48.9 controls: 49.8	age, sex, hearing	varying degrees of hearing, but matched between groups	excluded	gold cup electrodes were positioned at high forehead (active electrode), right and left ear lobes (reference), and low forehead (ground)	1000 Hz tone bursts presented at a rate of 1.1/s	enhanced N1 amplitude
Han et al., 2017 [108]	33 tinnitus ears 63 control ears	idiopathic	tinnitus: 38.7 controls: 37.7	age, sex, tinnitus ears,, hearing	≤25 dB HL at 0.5, 1, 2, and 3 kHz, and hearing threshold ≤40 dB HL at all frequencies	excluded	2-channel AgCI electrodes; Cz (reference); A1 and A2 (active and ground)	the first 250 ms was a 1 kHz tone followed by 250 ms of 8 kHz or 4 kHz pure tone	the normalized amplitude of the ACC of 8 KHz (tinnitus frequency) in tinnitus group was less than 4 and 8 kHz in normal control group
Mannarelli et al. 2017 [92]	20 tinnitus 20 controls	idiopathic	tinnitus: 50.1 controls: 49.4	age, sex, education	PTA <20 dB HL (up to 2000 Hz) PTA <30 dB HL (>2000 Hz)	excluded	9 central channels; referred to linked mastoids; Fpz (ground)	auditory oddball paradigm, tone bursts at 80 dB SPL, in the frequencies of 0.5 kHz (frequent stimulus) and 1 kHz (rare stimulus)	lower P3a amplitudes, prolonged N1 latency
Asadpour et al., 2018 [100]	15 tinnitus 6 controls	not mentioned	tinnitus: 39 controls: 27	hearing	normal hearing	not mentioned	32 EEG electrodes cap; tip of nose (reference)	auditory/visual oddball paradigm, auditory stimuli: tone bursts at 70 dB SPL, in the frequencies of 4 kHz (standard stimulus) and 6 kHz (rare stimulus) visual stimuli: 160 blue triangles as standard and 40 yellow circles as target stimuli	lower amplitude of auditory P300 peak in three EEG channels
Wang et al., 2018 [114]	95 mild tinnitus group, 112 severe tinnitus	idiopathic	mild tinnitus group: 47.88, severe tinnitus group: 48.18	not mentioned	≤25 dB HL (0.125–8 kHz)	excluded	Electrodes recorded at Fz, Cz, and Pz; A1 (left ear) and A2 (right ear) (reference electrodes)	auditory oddball paradigm, tone bursts at 85 dB HL, in the frequencies of 2 kHz (target stimulus) and 60 dB HL in 1 kHz (non-target stimulus)	compared with mild tinnitus patients, severe tinnitus patients exhibited longer P300 and N2 latencies
Campbell et al., 2019 [117]	21 tinnitus 45 controls	not mentioned	tinnitus: 21.51 (median) controls: 23.43 (median)	age, hearing	<15 dB HL (0.25–8 kHz) extended high-frequency (up to 16 kHz) was tested	tinnitus (*n* = 3) controls (*n* = 6)	128-channel electrodes net	gating paradigmtone 50 dB HL 250 Hz	no significant differences for P1, N1 or P2 amplitude
Durai et al., 2019 [95]	16 tinnitus 14 controls	idiopathic	tinnitus: 53.44 controls: 50.25	age, sex, hearing	matched (0.25–8 kHz)	excluded	66 active surface electrodes	ABA streaming paradigm, prediction paradigm	enhanced N1c, decreased P2 waveforms for frequency-4, and enhanced P2 waveforms for frequency-7 conditions
Jacquemin et al., 2019 [60]	22 tinnitus	heterogeneous	tinnitus: 51		heterogeneous	not mentioned	31 electrodes cap; chin (reference); right mastoid (ground); recorded at the right eye	auditory oddball paradigm, in the frequencies of 1 kHz (frequent stimulus) and 2 kHz (rare stimulus)	shortening of the N1, P2, N2, and P3 latencies after HD-tDCS treatment, the amplitude of N2 being significantly larger after HD-tDCS
Majhi et al., 2019 [103]	55 tinnitus 51 controls	idiopathic	tinnitus: 42.91 controls: 41.63	age, sex, education	Sensorineural hearing loss was classified into mild: 26–40 dB, moderate: 41–60 dB, severe: >61 dB	excluded	not mentioned	oddball paradigm	increased P300 latency and decreased P300 amplitude were found in sensorineural hearing loss with tinnitus cases. Increasing severity of tinnitus and degree of hearing loss
Sedley et al., 2019 [109]	26 chronic tinnitus, 26 non-tinnitus controls, 15 acute tinnitus)	not mentioned	chronic tinnitus: 55.4, controls: 59.7, acute tinnitus: 53.8	age, hearing	matched	not mentioned	64 channels	MMN paradigm	tinnitus subjects had larger responses to upward deviants and less responses to downward deviants than matched controls
Vasudevan et al., 2019 [96]	10 tinnitus 10 controls	not mentioned	tinnitus: 38.8 controls: 37.9	age, sex, hearing	≤40 dB HL (500 Hz, 1 kHz, and 2 kHz)	excluded	32-channel EazyCap; combined mastoid (reference)	auditory oddball paradigm, tone bursts at 75 dB SPL, in the frequencies of 1 kHz (standard stimulus) and 1.5 kHz (deviant stimulus)	larger N1 and P3 amplitudes along with prolonged P3 latency
Mohan et al., 2022 [105]	10 tinnitus 10 controls	idiopathic	tinnitus: 25.9 controls: 27	age, hearing	≤30 dB HL (0.25–8 kHz)	excluded	64-channel EazyCap; Cz (reference)	auditory oddball paradigm	increased P300 amplitude

### 2.3. Gap Detection

#### 2.3.1. Background

As an objective method to detect the presence of tinnitus, the gap-prepulse inhibition of the acoustic startle (GPIAS) was generally used in animals [118,119,120]. A silent gap embedded in a background noise before the startling sound can inhibit the acoustic startle reflex and when tinnitus fills in the gap, impairment of gap inhibition will be present. The principle of this theory is dependent on the assumption that tinnitus can fill in the silent gaps of background sound and alter the gap response of animals, which is presented as a ratio between the magnitude of the startle stimulus without the gap (no-gap trail) and a silent gap preceding continuous narrowband noise (gap trails) [121]. New methods were presented according to the fact that the gap-filling hypothesis may also apply to humans. In animals, the acoustic response startle may be measured based on the ear flick or pinna reflex [120], gap-induced reductions in evoked potentials of the auditory cortex [122] or a transducer in the cage floor to detect the “jump” of animals [121]. There are fewer human experiments on gap detection than animal experiments. The characteristics of these studies on gap detection are summarized in Table 3. In one study, the eye-blink reflex was used in humans to detect the gap [123]. Unfortunately, both the response to 50 ms gap-embedded stimuli with a high (similar to tinnitus pitch) and low frequency showed a deficit in gap detection ability using the eye-blink reflex in a study, which may be due to abnormal cortical auditory processing in patients with tinnitus [124]. Furthermore, Wilson et al. suggested that post-auricular muscle response is a sensitive method for measuring GPIAS [125]. Two experiments that used behavioral methods and background stimuli with narrow-band noise to detect gap recognition ability did not find differences in detection ability between the tinnitus group and the control group. However, the gaps they used were not the same, one was 50 ms, the other was increased or decreased from 30 ms, and there was heterogeneity in the hearing status of the tinnitus group in both studies [126,127]. Other experiments using white noise background stimuli with normal hearing in the tinnitus group found that tinnitus patients required longer gap durations to be detected [128,129,130,131]. Moreover, an experiment compared the amplitude of eye blinks with a background stimulus of narrow-band noise and found that the amplitude of the tinnitus group was higher than the control group, while the gap duration used in this experiment was 100 ms [132]. One study [118] showed, in both 500 and 4 kHz background noise frequencies, that a GPIAS deficit was present in tinnitus patients. This raised doubt regarding the opinion that tinnitus “fills-in” the gap, along with other studies which showed that the tinnitus and background noise frequencies were uncorrelated [49,133]. It may be partially a result of the existing technology for testing tinnitus pitch, which is not mature enough, as well as the multiple nature of tinnitus, such as narrowband noise, pure tone, white noise, broadband noise or a combination of the above [134]. One study considered that the tinnitus percept does not fill in the silent gap. However, they cannot exclude the possibility of the interference of pre-attentive filtering of sensory stimuli in the GPIAS sensorimotor gating paradigm [126]. In addition, the gap inhibition of the startle reflex can be detected in the cortical potential of patients with subjects who were given tinnitus-like sounds [135]. This contributes to the exploration of whether these responses can be masked by tinnitus, which further supports the reliability of GPIAS in evaluating tinnitus.

#### 2.3.2. Gap Detection Studies

Traditional gap detection is not an electrophysiological approach, and here we focus on gap evoked cortical potentials. Different conclusions were raised from different studies on whether gap detection is impaired in tinnitus patients using psychophysical methods. Since behavioral responses are more influenced by intention in humans, the current measurement in humans mainly focused on recordings of auditory cortical responses, which may objectively provide a further understanding of the association between tinnitus and alterations in gap processing, rather than behavioral gap detection [134]. Suh et al. considered that auditory cortical responses are more reliable and objective than behavioral responses in humans. Moreover, they found that the inhibition rate of N1–P2 amplitude in normal people was the highest when the gap was embedded 20 ms before 1 kHz of startle stimulation without Hanning window, and the background noise was 20 dB HL, which was the optimal stimulus [142]. Few studies used the gap in noise paradigm to detect gap-elicited evoked potentials. The gap in the noise test is composed of silent intervals or gaps embedded in a series of segments of broadband noise [143]. The cortical auditory evoked potential, which can be recorded by electroencephalography (EEG), mainly shown by the P1–N1–P2 waveform complex, can be evoked by the presence of a silent gap in background noise [134]. A study used this paradigm in humans to determine whether cortical auditory evoked potentials revealed differences in gap management between the tinnitus group and the control group, and found that the P1–N1–P2 area and amplitude evoked by the gap varied with the duration of the gap. However, contrary to speculation, there was no significant difference in peak amplitude and latency between the tinnitus group and the control group. This may be related to the absence of a specific age and the degree of hearing loss, the identification of specific subgroups of tinnitus, and the exclusion of hyperacusis [134].

Gap evoked N1, which is generated mostly in temporo-parietal areas, was present at the auditory cortex [144]. One study [139] used the N1–P2 complex to investigate whether it can assess tinnitus depending on the gap-prepulse inhibition (GPI) paradigm in tonal 8 kHz patients with tinnitus and controls. However, it found that the tinnitus group only exhibited an inhibitory deficit of the N1–P2 complex under 20 ms interval conditions with tinnitus-pitch-matched frequency background noise. There was no correspondence between the background frequency and the tinnitus-pitch, which was presumably affected by stimulus properties and higher cognitive processing. Another study [145] found that the neural GPI ratio (gap/no gap) using the N1–P2 amplitude was affected by the gap duration and background frequency, and there is a lower effect of age on the specific gap duration. Therefore, an appropriate combination of gap duration and background frequency will enhance the sensitivity and specificity and decrease the influence of confounding factors, such as age, when tinnitus is assessed with the GPI paradigm. Several studies suggested that gap duration should be less than 50 ms, thus the auditory cortex is necessary for perceptual gap detection. Otherwise, non-cortical areas, such as the brainstem will be involved to mediate longer gap detection. Meanwhile, studies have found that tinnitus patients with normal hearing thresholds need longer gaps than controls due to the difficulty in auditory temporal resolution [128]. Therefore, the duration must be higher than the gap detection threshold [146,147]. However, few researchers agree that the impairment of GPIAS can be affected by more peripheral pathways through the cochlea, brainstem, and inferior colliculus, but not by the cortical response [148]. Furthermore, Fournier et al. found that monaural and binaural stimulation can affect the GPI results and recommended monaural presentation as the best-suited application to detect tinnitus [136]. 

In addition, both studies using the 7 ms gap duration and pure tone background stimuli [137,140] found that the amplitude and area under the curve of MMN for silent gap deviants in humans will reduce in the decompensated tinnitus group or tinnitus group compared with controls, which may result from the gap detection deficit in tinnitus. Another study using the 15 ms gap duration and pure tone background stimuli found decreased MMN amplitudes in the tinnitus group [141].

#### 2.3.3. Main Findings

Factors, such as sex, age, and hearing conditions have an impact on the gap process [145,149,150,151]. In future research, attention should still be paid to matching these, and intra-group differences should be controlled as much as possible to reduce the degree of dispersion. Moreover, gap detection ability was affected by different aspects of tinnitus severity when grouped by THI [131]. The hyperacusis or anxiety that tinnitus patients often accompany will make the patient more responsive to startle stimuli, which may ultimately affect the experimental results [124]. Hearing loss reduces the patient’s temporal resolution and requires the use of longer gaps to cancel the effect. Although the use of long gaps (>30 ms) in the absence of significant hearing loss (>60 dB) has little or no effect on the results, it is best to match hearing levels in future experiments [126]. Pre-pulse inhibition of narrowband noise is more effective than pure tone noise. In addition, it can reduce the experiment time to prevent short-term habituation [139,152,153]. Overall, the etiology, the characteristics of tinnitus and background noise as well as its presentation mode, the frequency and loudness of stimuli, the age and race of experimental subjects, tinnitus matching procedures, and detection methods, such as behavior tests and cortical measures or combinations of sub-cortical measures, all affect the outcome. Furthermore, in future studies, these considerations may need to be taken into account to determine whether tinnitus can fill in the silent gap and whether it can be assessed by this hypothesis or not.

### 2.4. EcochG 

#### 2.4.1. Components

EcochG is a method of recording cochlear and auditory nerve population potentials in response to sound [154] from electrodes in the round window, the promontory of the human cochlea, the tympanic membrane (TM), the external ear canal or the cochlea. It contains four basic potentials: The cochlear microphonic (CM), the summating potential (SP), the auditory nerve neurophonic (ANN), and the compound action potential (CAP). Therefore, it can be a technique to diagnose cochlear synaptopathy, which is a factor contributing to tinnitus [155]. CM and SP are traditionally considered from hair cells, while CAP and ANN are considered to be produced by the auditory nerve. The CM is a potential arising to reflect the preserved cochlear hair cell activity. It almost exclusively arises from the currents that flow through the mechano-electrical transduction channels in the stereocilia of outer hair cells, in response to the basilar membrane movement with almost no contribution from inner hair cells [156,157]. CM recorded from the electrodes at the promontory and round window membrane almost all originate from the basal portions of the cochlea [158,159]. The threshold of the CM is mostly influenced by the quality of the recording apparatus, and the CM can easily be confused with an artefactual microphonic [160]. A direct current component resulting from the non-symmetric depolarization-hyperpolarization response of the cochlea is shown as SP, in which the polarity is highly dependent on the frequency and intensity of the stimulus [161], In addition, it is probably generated predominantly by the OHCs [159]. However, other studies conclude that a great contribution is made by the inner hair cells (IHCs) [162,163]. Recently, one study reported that not only IHCs and OHCs, but also a strong contribution of the auditory nerve, was confirmed in both gerbils and human beings with electrodes recorded at the round window [164]. Action potential (AP) reflects the potential of afferent cochlear nerve fibers as they enter the habenula perforate. According to the frequency of the stimulus, a particular cluster of nerve fibers will be recorded by EcochG. The click AP generated from the click stimulus can be derived from the entire nerve fibers of the cochlea, which is the algebraic sum of the individual AP [160]. The CAP induced by the tone pip and neural population responses that originate from different portions of the cochlea indicate the neural component in the EcochG, which reflects action potentials in auditory nerve fibers that synchronize with the onsets of the stimulus [165,166]. The delayed and long first spike latency distribution leads to low spontaneous rate fibers with little to no contribution to CAP, which mostly reflects the contribution of high- and medium-spontaneous rate fibers. One study has shown that the CAP amplitude and threshold can also be used to detect the damage of IHC since type I auditory nerve fibers can be influenced by the release of excitatory neurotransmitters from the IHC [163]. ANN, an ongoing response following the waveform of tonal stimulation, can provide information on temporal processing, which is derived from the phase-locked responses of auditory nerve fibers [167]. Recently, one study has shown that a mixture of CM and ANN was recorded in response to low frequencies in human subjects [168] and CM is phase-locked at all tone frequencies, while ANN is strong only at frequencies below 2000 Hz [169]. The ANN can be described as the convolution of a unit potential and the cumulative post-stimulus time histogram of auditory nerve fibers responding to the stimulus [170]. EcochG can be divided into two types according to the location of the electrodes: Trans-tympanic (TT) in which the electrode is placed on the promontory or round window of the cochlea, and extra tympanic (ET), which is recorded with a non-invasive electrode in the external auditory canal or on TM. Compared with ET, the amplitudes of TT were higher, but the difference in latencies was not significant [171,172]. There is no significant difference in the SP/CAP ratio between TT and ET recordings [173]. A comparison between simultaneous ET and TT recordings with tone bust stimuli found that they provide primarily the same information, while ET recordings appear to be less sensitive and require more stimuli to be averaged [174]. In addition, the location of electrodes on the tympanic membrane made a difference in the amplitude of AP and SP, while SP/AP ratios were comparable [175]. ECochG is gradually replaced by ABR, which is a non-invasive hearing test. However, it remains the method of choice to measure audiometry objectively under anesthesia [154]. Furthermore, the TT ECochG, unlike electrodes placed at a distance from the generators in ABR, can be recorded by electrodes that pass through the tympanic membrane to contact the promontory wall. Therefore, receptor potentials (CM and SP) and auditory nerve activity (CAP) can be separately analyzed efficiently by near-field recordings [176]. The non-invasive ET-ECochG procedure is more suitable to be carried out in humans compared with TT-ECochG in clinical applications. Previous studies of the application of EcochG mostly focused on diagnosing Ménière’s disease, exploring its relationship with auditory synaptopathy/neuropathy and intracochlear electrocochleography using cochlear implant electrodes more recently, while few studies explored the relationship between tinnitus and EcochG, in particular. 

#### 2.4.2. Previous Studies

It has been suggested that tinnitus may result from or be accompanied by potential cochlear synaptopathy, even in patients with normal hearing [32,177]. Subjects of normal hearing with tinnitus showed an increase in the SP/AP ratio in both ears compared with the control group of normal hearing without tinnitus by placing electrodes in the ear canal, indicating cochlear synaptopathy in tinnitus patients with normal hearing [177]. Severe IHCs damage in certain cochlear regions generally occurs in tinnitus patients regardless of whether a measurable hearing loss exists [155,178]. After treatment, the reduction of latency of CAP was statistically significant, which indicates a robust and synchronized firing of the neuron fibers, along with the improvement of the self-report tinnitus questionnaire scores among tinnitus patients. However, SP/AP ratios did not change significantly in one study [179]. CAP amplitudes were suppressed and thresholds increased in tinnitus patients induced by sodium salicylate in one study [180]. Improvement of CAP amplitude after low-level laser treatment in tinnitus subjects was shown in one study, which may indicate the reduction in neural network activity in the presence of tinnitus [181]. AP latency was prolonged significantly along with the amplitude of AP tending to reduction without significant difference in cases with tinnitus reduced after injection of lidocaine [182]. Overexposure to a loud sound will result in spontaneous hyperactivity, which has been considered a substrate for tinnitus [183]. The CAP amplitudes increased significantly in patients whose tinnitus was suppressed after electrical promontory stimulation and the enhanced amplitude probably resulted from synchronizing discharges of the auditory nerve fibers [184]. Overall, the CAP amplitude tends to decrease in patients with tinnitus in the majority of studies. However, to date, there are few studies on how the components of electrocochleogram change during tinnitus, and the majority of studies use electrocochleogram to supplement some data when studying other methods [182]. Moreover, some indirect evidence, such as EcochG can be used to detect synaptopathy, and synaptopathy can cause tinnitus [32,177]. After some time of treatment, tinnitus is relieved and the electrocochleogram is also changed [179,181,182]. These pieces of evidence show that electrocochleogram has the potential to detect tinnitus, but more in-depth research needs to be carried out in the future.

#### 2.4.3. Main Findings

Unfortunately, to date, there is no clear study on the specific changes of electrocochleogram during tinnitus and no standardized normal range of component potentials. In addition, there is great heterogeneity among the studies due to the difference in location of electrodes, recording instrument, recording standard, the subtype of tinnitus, etc. in different researches. Whether the electrocochleogram can objectively evaluate the existence of tinnitus still needs to be explored. It is a promising research direction due to its near-field characteristics, which will lead to the provision of more detailed information and generation of more robust waveforms, in order that we can observe more minor changes, particularly the intracochlear EcochG. However, invasiveness may be a major obstacle to its clinical application.

## 3. Discussion

At present, self-reports or some questionnaires are mainly used to detect tinnitus in clinic. Imaging detection will be used in subjective tinnitus with asymmetric or neurological symptoms. In the case of paroxysmal tinnitus, EEG and ABR can be used to diagnose whether there is epilepsy or compression symptoms. If MRI is not available, ABR can be selected when tinnitus patients are accompanied by deafness or vertigo. Moreover, ABR or EEG can be used for post-traumatic tinnitus. EcochG is a sensitive indicator for endolyphatic hydrops. However, the above-mentioned electrophysiological methods are rarely used to detect tinnitus in clinic. The main reason is that no electrophysiological indicator with high sensitivity and specificity has been found at present.

**Figure 1:**
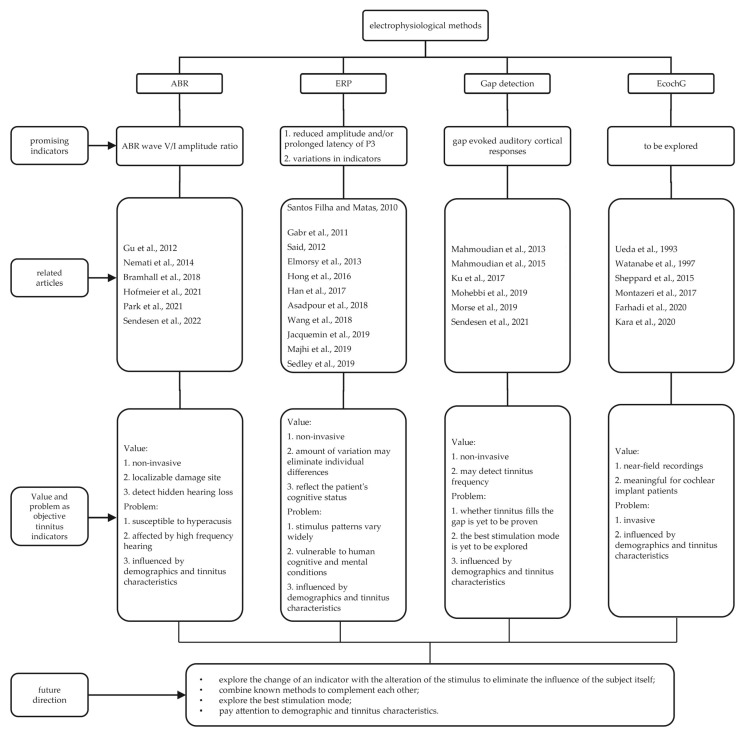
Flow chart of full-text results [30,31,32,34,44,60,87,91,93,99,100,101,103,104,108,109,114,134,137,138,139,140,141,177,179,180,181,182,184].

Figure 1 shows a general summary of various detection methods. Research regarding the objective detection of tinnitus is still faced with great difficulties and challenges, with several disputes and little consensus. In general, there is a lack of recognized objective measurement methods for tinnitus. Without this method, we cannot objectively judge the efficacy of drugs, and any future research on tinnitus will be seriously affected without these measures. From the perspective of treatment, the objective detection of tinnitus is the key for clinicians and patients to understand the progress and effectiveness of treatment. Reduced wave I amplitude and enhanced wave V/I amplitude ratio in ABR in tinnitus patients were widely accepted in numerous studies. However, several recent studies have come to the opposite conclusion, namely, that previous findings may be mistaken by co-occurrence of hyperacusis or that hidden hearing loss and elevated activity in the central auditory system may be uncorrelated with tinnitus [33,34,51,185]. Moreover, the objective diagnostic value of other methods for tinnitus is controversial. In the case of ERP, the degree of variation of each component varies greatly among different individuals, and few studies have indicated a new direction for us. 

As mentioned above, the heterogeneity between different articles is largely due to different experimental parameters and experimental populations. The etiology of tinnitus, the demographic characteristics of the included population, whether there are other diseases, the definition of normal hearing, whether high-frequency hearing is measured, and the location of the electrodes, etc., can all affect the experimental results. In the future, the detection performance of a certain index on a specific subtype of tinnitus under a specific stimulation mode can be carried out to find a suitable method for different populations. In addition, it is important to explore which stimulus method is more efficient for detection.

A major obstacle to the development of objective tinnitus electrophysiological testing methods is the overlap of this symptom with other symptoms, such as hearing loss, hyperacusis, and emotional symptoms. Therefore, we can explore the change of one component in an individual to eliminate other influencing factors. These two studies found that there is a fair degree of efficacy in detecting tinnitus using the upward and downward variation of MMN and the changes of N1–P2 under different stimuli [108,109]. In addition, the results of these studies are still a long way from translation into clinical applications. There is certainly a potential change recorded by the electrodes during tinnitus. The heterogeneity of the results is largely due to discrepancies between previous studies, such as induction of tinnitus in animal experiments, etiology, and classification of tinnitus in humans, immaturity of pitch matching methods, the accuracy of the instrument, characteristics of stimulation, demographic characteristics of experimental subjects etc. Therefore, future research in this area is essential to define general criteria and refine the research direction. In the gap detection section, we mainly focus on gap-induced ERP, which is equivalent to combining gap detection with ERP. In addition, one study [186] used ABR to detect gaps and the results showed a significant difference only in the range near the tinnitus frequency. This provides us with a clue that in the future, we can combine known methods to objectively detect tinnitus for the attainment of more sensitive and specific indicators.

## Figures and Tables

**Table 3:** Characteristics of gap detection studies, including demography, stimulation patterns, and outcomes.

Author, Year	Groups and Number	Etiology of Tinnitus	Mean Age	Matched	Hearing Status	Stimuli	Measuring Method	Outcome
Sanches et al., 2010 [130]	20 tinnitus 28 controls	not mentioned	tinnitus: 33.8 controls: 28.8	not mentioned	≤25 dB HL (0.25–8 kHz)	2, 3, 4, 5, 6, 8, 10, 12, 15 or 20 ms gap embedded in 50 dB SL white noise	threshold and number of correct responses	lower percentage of correct responses and longer time interval required to detect gap in the tinnitus group
Campolo et al., 2013 [126]	13 tinnitus 13 controls	not mentioned	tinnitus: 50 controls: 24	not mentioned	tinnitus: varying degree of HLcontrols: ≤20 dB HL (0.25–8 kHz), ≤35 dB HL above 8 kHz	tinnitus: 50 ms silent gaps embedded in one-third octave bands of noise located 1-octave below, 1-octave above, and at the pitch of the subject’s tinnitus controls: 50 ms gaps embedded in NBN located at 1.2, 8, and 12.6 kHz	press the response button within 2s if they detect a gap	both tinnitus and controls could detect the 50 ms gaps
Fournier et al., 2013 [136]	test: 15 tinnitus 17 controls retest: 10 tinnitus 9 controls	not mentioned	test-tinnitus: 28.5 controls: 23 retest- tinnitus: 29.3 controls: 4.5	sex, education	<35 dB HL (0.25–4 kHz)	Startle noises were 50 ms broadband noise bursts (20 Hz–20 kHz) set at 105 dB SPL backgroundnoise set at 65 dB SPL The low-frequency background noise was centered at 500 Hz (200–1200 Hz) and high-frequency background noise at 4 kHz (3.5–4.5 kHz), 50 ms silent gap presented 120 ms before the startle sound	eyeblink: % inhibition = [(pulse-alone) − (gap/prepulse)]/(pulse-alone) × 100.	normal prepulse inhibition but higher reactivity to the startle sounds in the tinnitus group, the tinnitus group displayed a consistent deficit in gap processing at both low- and high-background noise frequencies
Mahmoudian et al., 2013 [137]	28 tinnitus 33 controls	idiopathic	tinnitus: 33.78 controls: 35.21	age, sex	≤20 dB HL (0.25–2 kHz), ≤40 dB HL (4–8 kHz)	7 ms silent gap embedded in standard stimuli presented at an intensity of 65 dB SPL pure tones of 0.5, 1, and 1.5 kHz	electroencephalogram (EEG)	reduced MMN amplitude and area under the curve for the silent gap in tinnitus group
Mehdizade et al., 2013 [128]	20 tinnitus 20 controls	not mentioned	tinnitus: 30.31 controls: 27.8	age, sex	≤20 dB HL (0.25–8.0 KHz for air conduction and 0.25–4.0 KHz for bone conduction)	0 to 3 silence gaps of different durations (2–6, 8, 10, 12, 15, 20 ms) embedded in 6s 50 dB SL white noise stimuli	identify the silence gaps	tinnitus patients needed a longer duration of gap to detect than those of the non-tinnitus subjects
Jain and Sahoo, 2014 [131]	10 mild tinnitus 10 moderate tinnitus20 controls	idiopathic	mild tinnitus: 36.8 moderate tinnitus: 39.4 controls: 36.1	age	≤25 dB HL (0.25–8 kHz)	a temporal gap embedded in 500 ms broadband noise	three-interval, alternate forced-choice (3-AFC) method	individuals with moderate tinnitus need larger silent intervals to detect a gap within a noise than individuals with mild tinnitus as well as those without complaints of tinnitus
Shadwick& Sun, 2014 [132]	7 tinnitus 9 controls	not mentioned	range: 20–55	age	controls: ≤20 dB HL (0.25–8.0 KHz)	The background noise was a narrowband noise with a 100 Hz bandwidth presented at 38–40 dB SPL centered at a frequency of the patient’s tinnitus The startle noise was a broadband signal at 100 dB SPL The gap duration was 100 ms and the duration of startle stimulus was 50 ms (rise/fall time 1 ms) ten trials at each frequency (0.5–8 kHz)	eye-blink amplitude	The amplitude of the startle response in the tinnitus group with normal hearing thresholds was significantly higher than the control group and those with tinnitus and hearing loss
Boyen et al., 2015 [127]	22 tinnitus 20 nontinnitus 10 controls with normal hearing	not mentioned	tinnitus: 53 non-tinnitus: 52controls: 23	tinnitus and non-tinnitus: age, gender, and hearing characteristics matched	Threshold differences between ears were 20 dB or less for at least five of the six test frequencies (0.25–8 kHz) controls: ≤20 dB HL (0.25–8 kHz)	Four 300 ms narrow-band noise (4–8, 4–5, 5–6.3, 6.3–8 kHz) served as stimuli and were presented at 5, 10, and 25 dB SPL above their respective hearing thresholds The gap size at the start of the test was 30 ms	two-down/one-up adaptive procedure (2D1U)	tinnitus group did not display elevated gap thresholds
Mahmoudian et al., 2015 [138]	28 tinnitus	idiopathic	tinnitus: 35.33		≤20 dB HL (0.25–2 kHz), ≤40 dB HL (4–8 kHz)	7 ms silent gap embedded in standard stimuli presented at an intensity of 65 dB SPL pure tones of 0.5, 1, and 1.5 kHz	EEG	No statistically significant differences in MMN amplitude and AUC of gap after AES treatment
Ku et al., 2017 [139]	16 tinnitus 18 controls	not mentioned	tinnitus: 59.2 controls: 59.2	age, hearing	<70, 30, and 70 dB HL at 0.5, 1, and 8 kHz frequencies, respectively	20 dB SL continuous pure tone (8 kHz or 600 Hz) background noise and a 65 dB SL intense sound stimulus (1-kHz tone burst of 20-ms duration) 100-, 50- or 20-ms temporal gaps	the peak-to-peak amplitude of the N1–P2 complex in response to the gap-intense sound stimuli/peak-to-peak amplitude of the N1–P2 complex in response to the no-gap-intense sound stimuli	GPI deficit of patients with tinnitus was found on the N1–P2 complex with the tinnitus-pitch-matched frequency background noise and 20-ms gap duration
Mohebbi et al., 2019 [140]	20 compensated tinnitus 20 decompensated tinntitus 20 controls	not mentioned	compensated tinnitus: 44.35 decompensated tinntitus: 42.35 controls: 40.05	age, hearing	≤20 dB HL (0.25–2 kHz), ≤40 dB HL (4–8 kHz)	7 ms silent gap embedded in standard stimuli presented at an intensity of 85 dB SPL pure tones of 7, 8, and 8.5 kHz	EEG	reduced MMN amplitude and area under the curve for the silent gap deviant in decompensated tinnitus group compared with normal control and compensated tinnitus group
Morse et al., 2019 [134]	13 tinnitus 13 controls	not mentioned	tinnitus: 52.85 controls: 54.54	age, sex, hearing	mean PTA tinnitus: 18.87 controls: 20.15 (0.25–8 kHz)	6-s white noise segments with one to three silent gaps embedded. gaps ranging in duration between 2 and 20 ms	behavioral gap detection threshold: pressing a button each time a silent gap was perceived. Gap evoked P1–N1–P2 amplitude, latency, and area	no significant difference in silent gap evoked P1–N1–P2 amplitude, latency or area differences between groups
Sendesen et al., 2021 [141]	16 tinnitus 20 controls	idiopathic	tinnitus: 28.5 controls: 27.9	age, sex, hearing	<20 dB HL (0.125–16 kHz)	15 ms silent gap embedded in standard stimuli presented at an intensity of 65 dB SPL pure tones of 0.5, 1, and 1.5 kHz	EEG	reduced MMN amplitude for the silent gap in tinnitus group. no statistically significant differences for MMN latencies between the groups
Raj-Koziak et al., 2022 [129]	54 tinnitus 43 controls	not mentioned	tinnitus: 37.1 controls: 35.5	hearing	≤20 dB HL (0.125–8 kHz) High-frequency (9–16 kHz) was tested	gap with a duration of 10 ms and decreased or increased by 50% embedded in 50 dB HL white noise	detect gap	tinnitus patients needed a longer duration of gap to detect than those of the non-tinnitus subjects

## Data Availability

Not applicable.
